# Contrasting sea ice conditions shape microbial food webs in Hudson Bay (Canadian Arctic)

**DOI:** 10.1038/s43705-022-00192-7

**Published:** 2022-10-23

**Authors:** Loïc Jacquemot, Adrien Vigneron, Jean-Éric Tremblay, Connie Lovejoy

**Affiliations:** 1 Département de Biologie, Université Laval, Québec, QC Canada; 2 Institut de Biologie Intégrative et des Systèmes (IBIS), Université Laval, Québec, QC Canada

**Keywords:** Small RNAs, Microbial ecology, Biodiversity, Ecosystem ecology, Climate-change impacts

## Abstract

The transition from ice-covered to open water is a recurring feature of the Arctic and sub-Arctic, but microbial diversity and cascading effects on the microbial food webs is poorly known. Here, we investigated microbial eukaryote, bacterial and archaeal communities in Hudson Bay (sub-Arctic, Canada) under sea-ice cover and open waters conditions. Co-occurrence networks revealed a <3 µm pico‒phytoplankton-based food web under the ice and a >3 µm nano‒microphytoplankton-based food web in the open waters. The ice-edge communities were characteristic of post-bloom conditions with high proportions of the picophytoplankton *Micromonas* and *Bathycoccus*. Nano‒ to micro‒phytoplankton and ice associated diatoms were detected throughout the water column, with the sympagic *Melosira arctica* exclusive to ice-covered central Hudson Bay and *Thalassiosira* in open northwestern Hudson Bay. Heterotrophic microbial eukaryotes and prokaryotes also differed by ice-state, suggesting a linkage between microbes at depth and surface phytoplankton bloom state. The findings suggest that a longer open water season may favor the establishment of a large phytoplankton-based food web at the subsurface chlorophyll maxima (SCM), increasing carbon export from pelagic diatoms to deeper waters and affect higher trophic levels in the deep Hudson Bay.

## Introduction

Over the last 30 years, the Arctic has experienced drastic changes in summer ice cover and extent [1, 2]. These changes are particularly marked in the Hudson Bay (HB), an Arctic to sub-Arctic inland sea which transitions from first-year ice cover in winter to open ocean conditions in summer, a likely situation for the entire Arctic Ocean in the near future [3, 4]. Long term trends in sea ice formation and spring melt in HB show that the ice-free season increased by more than 3 weeks between 1981 and 2010 [5]. Future scenarios for the HB predict that increasing sea surface temperature and freshwater inputs through precipitation and river discharge, will result in continued lengthening of the open water season [6, 7], with consequences for diversity and functional roles of microbial plankton communities [8, 9].

The timing of spring phytoplankton blooms, which account for the annual peak in primary production in the Arctic, is closely related to ice conditions [10] and ongoing observations show that the bloom is occurring earlier in Arctic regions [11, 12]. In recent years, this peak has been in the marginal ice zone in May-June during the ice break up in HB [13]. This is followed by the formation of subsurface chlorophyll maxima (SCM) in the open water [14], which tend to persist in the summer and autumn [15, 16]. Influenced by prevailing winds, the variability in ice conditions in spring creates large spatiotemporal patterns in primary production between central and northwestern HB [14].

Phytoplankton blooms are characterized by a succession of species governed by their affinity to light and nutrients [17, 18]. Seasonal patterns in ciliates and dinoflagellates (single celled microzooplankton) are also evident as they closely follow their phytoplankton prey [19]. Moreover, the organic matter released during phytoplankton blooms and during the subsequent bloom collapse, provide a series of ecological niches for specialized communities of heterotrophic bacteria and microbial bacterivores [20–22]. The dissolved organic matter (DOM) released by phytoplankton is a source of high-quality substrate [23–25] that sustains bacterial activity and diversity [26–28]. Although the spring phytoplankton bloom associated with the sea ice melt in HB has been documented, the potential cascading effects of ice retreat on microbial food webs have not been explored to date. This is of critical importance for understanding and predicting future HB and Arctic Ocean ecology since a modification of the microbial food web can alter nutrient recycling and export of organic material to the depth [29].

The spring sea ice breakup in HB begins with early opening in the northwestern region and progresses toward the center of the bay under the influence of northwesterly winds [30, 31]. The spring-to-summer transition is then a critical period when loss of ice caused by melting or drifting controls access to light and influences nutrient concentrations needed by phytoplankton. To investigate the microbial community dynamics of the Hudson Bay during ice breakup, we collected samples from June 2018 from northwestern to more central HB corresponding to a gradient of increasing ice cover. High throughput sequencing of the V4 region of 18 S rRNA (eukaryotes) and 16 S rRNA (Archaea and Bacteria) was carried out to identify the taxonomic composition of microbial communities. We then used co-occurrence networks to investigate the response of the microbial food web to the sea ice retreat. Our working hypothesis was that surface microbial community distribution influenced by ice concentrations could affect microbial systems in the deeper waters, with potential implications for carbon and energy export to the shallow benthos in HB.

## Material and methods

### Metadata

Total Sea Ice Concentration (SIC) data were obtained from the Canadian ice service digital Archive, which records daily ice charts in Hudson Bay [32]. When the exact date did not match our sampling day, we used the mean of the two surrounding closest dates. All field work was carried out aboard the Research Icebreaker CCGS *Amundsen* in June 2018 as part of the Hudson Bay System Study (BaySys) [33]. Conductivity, Temperature and Depth (CTD) profiles were taken using a Sea-Bird SBE-911 (Sea-Bird Scientific, Bellevue, WA USA) profiler mounted on a rosette also equipped with a dissolved oxygen (Sea-Bird SBE-43), chlorophyll fluorescence (Seapoint Sensors Inc., Exeter, NH), fluorescent colored dissolved organic matter (CDOM; Wetlabs ECO, Philomath, OR, USA), and transmissometer (WETlabs C-Star, Sea-Bird Scientific) sensors. The dissolved oxygen sensor was calibrated onboard against Winkler titrations.

Discrete water samples for nutrients, cell enumeration using Flow Cytometry (FCM), and nucleic acids were collected from 11 stations in the northwestern sector and central Hudson Bay. Water was collected directly from 12 L-Niskin-type bottles mounted on the rosette system, with bottles closed on the upward cast. To investigate the vertical structure of microbial communities, we sampled three to four depths: the surface mixed layer, the subsurface chlorophyll maximum (SCM) layer, 70 meters and at 10 m from the bottom. The depth of the SCM was identified on the downward cast from the Chl *a* in situ fluorescence peak. When stations were shallow, three depths were collected (with no 70 m sample collected). A total of 42 water samples were analysed (Supplementary Table S1).

For nucleic acids, following prefiltration with a 50 µm mesh, to reduce mesozooplankton in the samples, six liters of water was sequentially filtered through 3-µm and 0.22-µm pore size filters as in [34]. Nutrients and FCM samples were collected from the same depths and sample bottles. Nitrate (NO_3_), nitrite (NO_2_), phosphate (PO_4_) and silicate (Si(OH)_4_) was measured following GEOTRACES protocols and analysed on board with a Bran-Luebbe 3 autoanalyzer [35]. All FCM samples were fixed in 1% (v/v) glutaraldehyde and stored in -80°C until laboratory analysis.

DNA and RNA samples were co-extracted from the filters using AllPrep DNA/RNA Mini kit (Qiagen, Hilden, Germany) following the suggested protocol as in [34]. The RNA was converted to complementary DNA (cDNA) using the High-Capacity Reverse Transcription Kit (ThermoFisher, USA). Absence of DNA contamination in the RNA extractions was confirmed by PCR. For eukaryotes, the V4 region of 18S rRNA gene (rDNA) and 18S rRNA (rRNA) was amplified to construct libraries using a combination of universal forward E572F and reverse primers E1009R [8]. For prokaryotes, the primer 515F-806R targeting the V4 region of 16S with was used [36]. Amplicons were purified then tagged for multiplexing with MiSeq® specific linking primers and equimolar concentrations of amplicons were pooled and sequenced on two Illumina MiSeq® runs by the “Plateforme d’Analyses Génomiques” (IBIS, Université Laval, Canada). Raw paired-end reads have been deposited in NCBI under BioProject accession numbers PRJNA627250 and PRJNA721720 for eukaryotes and prokaryotes, respectively.

### Flow cytometry

Microbial cell concentrations were measured on a BD Accuri^TM^ C6 flow cytometer (BD Biosciences, San Jose, CA). Total phytoplankton cell counts were estimated from chlorophyll red fluorescence (FL3) and forward-scattered light (FSC). Samples were run for 10 min at fast flow rate (66 µl/min). Bacterial cell counts were measured from separate aliquots stained with Sybr green (FL1) and FL3 and run for 5 min at slow flow rate (14 µl/min). Within the total phytoplankton gate, we defined three populations: Cyanobacteria were distinguished from pico‒ (<2 µm) and nano‒phytoplankton (>2 µm) based on orange phycoerythrin fluorescence (FL2). The pico‒ and nano‒phytoplankton populations with Chl *a* fluorescence were segregated based on FL3, FL2 and FSC (Supplementary Fig. S1a).

### Data analysis

Eukaryotes along with Bacteria and Archaea (referred to as prokaryotes) rRNA and rDNA were sequenced from the large (3–50 µm) and small (0.22–3 µm) fractions of the 42 water samples (Supplementary Table S1). Overlapping paired end reads from the fastq files were processed using DADA2 [37] within the qiime2 environment [38]. Removing of primers, denoising of low-quality reads, merging and removing of chimeras was performed using the denoise-paired command in DADA2. The two denoised runs were merged and taxonomy was assigned to each ASV in mothur using the PR2 database v4.12 [39] and SILVA 132 [40] for eukaryotes and prokaryotes, respectively. For the cross-comparisons between samples, small and large fraction communities were summed together, and sequences affiliated to Metazoa and chloroplasts as well as sequences at the unclassified Phylum level were removed from the analysis using the R package *Phyloseq* [41]. For relevant taxa, taxonomy was refined by BLASTn against the NCBI nr database.

To correct for differential sequencing depth, data were transformed to a relative abundance table. To reduce false positives, ASVs below the threshold of 1 × 10^−5^ total relative abundance were removed from the matrix table. For each individual sample, ASVs accounting for ≤ 0.003% of total relative abundance were removed. This resulted in a relative abundance table of 1371 ASVs for eukaryotic rDNA, 1384 ASVs for eukaryotic rRNA, 3891 ASVs for prokaryotic rDNA and 4152 ASVs for prokaryotic rRNA. ASV sequences were then aligned using MAFFT and the best-scoring maximum likelihood (ML) trees were selected among 100 trees constructed under the GTR + GAMMA model of substitution using RaXML [42]. All the subsequent clustering analysis were run on R using vegan packages [43]. For each relative abundance table, Bray–Curtis and GUniFrac matrices were calculated from Hellinger transformed data. Non-metric multidimensional scaling (NMDS) was performed on the Bray–Curtis and GUnifrac matrices using the cmdscale() function. Eukaryote and prokaryote matrices were compared using Procrustes analysis and Mantel tests. The significance of the m^2^ statistic resulting from the comparison of two matrices by orthogonal Procrustes analysis and Mantel significance value R^2^ was tested by 999 permutations.

Distance-based redundancy analysis (db-RDA) of microbial communities in rDNA and rRNA dataset was performed on the Bray–Curtis matrix with standardized environmental variables using the capscale() function. The adjusted R^2^ measures the unbiased amount of explained variation and was used to select significant variables using a forward selection and 9999 ANOVA permutations. Phosphate and silicate were removed from the final db-RDA calculation because of strong co-variation with nitrate. Z-scores (Z-score = ASV relative abundance − mean relative abundance/standard deviation) were calculated on the 50 most abundant ASVs based on their mean relative abundance in the rDNA dataset.

Co-occurrence networks were constructed with the 500 most abundant ASVs of both prokaryotes and eukaryotes from the rDNA relative abundance table using the CoNet plugin [44] in Cytoscape [45]. This threshold was selected based on a rank abundance curve to reduce the effect of very rare ASVs on correlation calculations (Supplementary Fig. S2). To minimize false-positive correlations between samples from the euphotic zone and the bottom, the analyses were run separately, first with samples from surface and SCM and then with samples from 70 m and the bottom. The deepest samples of the shallow (less 71 m deep) more inshore stations (st22 and st19) were removed from the analysis. Positive associations were inferred with four methods: Pearson’s product moment, Spearman’s rank correlation, mutual information (distance between probability distributions), and Bray–Curtis distance. To minimize sparsity effects, ASV rows with ≥5 null (0) values were removed from the analysis (row_minocc = 5). The initial threshold was selected such that the initial network contained the 1000 positive edges by all four measures. For each measure and edge, 1000 permutations and bootstrap scores were generated and measure-specific *p* value scores were merged using Brown’s method [46]. False-positives were detected and removed from the final network by applying Benjamini–Hochberg correction. Unstable edges with a score outside the 95% confidence interval defined by the bootstrap distribution were discarded. Only edges supported by at least two methods and with *p* value <0.01 were conserved in the final network.

## Results

### Environmental data

**Fig. 1:**
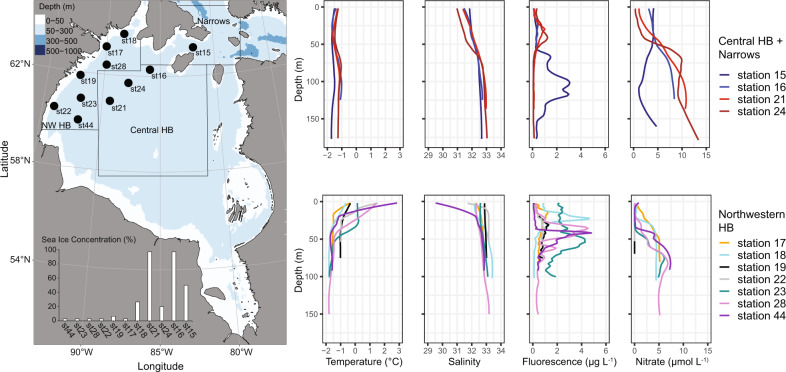
Water sampling sites and water column properties in the Hudson Bay. Left panel shows the sampling locations with sea ice concentrations. Right panel indicates vertical CTD profiles for temperature, salinity, Chl fluorescence and nitrate concentration down the water column.

At the time of sampling, stations st21 and st16 in central HB were effectivly completely covered by ice with 97 % surface sea ice concentrations, whereas st24 and st15 had sea ice concentrations between 20 and 50% characteristic of mobile ice pack (Fig. 1). Stations st19, st17, st22, st23, st44 and st28 in northwestern HB were ice-free. Temperature and salinity profiles showed cold (−1.3 to −1.4 °C) and moderate salinity (31.1–31.5) conditions in the surface layer under the ice in central HB (Fig. 1, Supplementary Table S1). By contrast, in northwestern HB surface waters were warmer, ranging from 0.1 to 2.4 °C. Salinity was heterogenous in northwestern HB. St44, which was sampled on 24 June, had the freshest surface waters (30.1) recorded in samples collected for this study. Bottom waters were uniformly saltier (31.5–32.6) and colder (−1.1 to −1.8 °C) compared to the surface waters. For nutrients, nitrate and silicate were low above 50 m in northwestern HB and higher concentrations were seen under the ice in central HB, with an upper water column nitrate maximum of 3.75 µmol L^‒1^ and a silicate maximum of 8.77 µmol L^‒1^ at the SCM at st6 (Fig. 1, Supplementary Table S1). By contrast, the 70 m and bottom layers were enriched in nitrate, phosphate and silicate with an average of 6.21, 1.08 and 14.33 µmol L^‒1^, respectively.

Concentrations of Chl *a* from the CTD fluorescence probe in the central HB were mostly below 1 µg L^-1^ and changed little down the water column with only a weak SCM (1.71 µg Chl *a* L^-1^) developing at st24 at 46 m (Fig. 1). A distinct SCM was evident in the open water stations, which was especially pronounced in the more offshore stations. The maximum Chl *a* fluorescence from the CTD at any water sample depth, was at the SCM of st28 (4.81 µg L^-1^) (Supplementary Table S1).

Nano (3–20 µm) and micro (>20 µm) phytoplankton cell concentrations ranged from 8 cells ml^-1^ at the bottom of st16 to 4.34 10^3^ cells ml^-1^ at the SCM of st44 (Supplementary Fig. S3, Table S1). At the surface, nano– and micro‒ phytoplankton cell concentrations showed no significant correlation with diatom ASVs relative abundance in the mix of diatoms and nano‒flagellates in this size category. The correlation was higher with only nano‒flagellate ASV relative abundance, but still not significant. Conversely, there was a significant linear correlation between the smallest taxa belonging to Haptophyta and Chlorophyta ASV relative abundance and pico‒phytoplankton cell concentrations from surface and SCM, with increased pico‒phytoplankton under the ice and a maximum of 1.54 10^5^ cells ml^-1^ at the SCM of st18. Bacteria (prokaryote) cells showed higher concentrations in the euphotic zone than in the deeper waters, ranging from 1.44 10^6^ cells ml^-1^ at the surface of st28 to 5.12 10^5^ cells ml^-1^ at the SCM of st23 (Supplementary Fig. S1).

### Structure of microbial communities

**Fig. 2:**
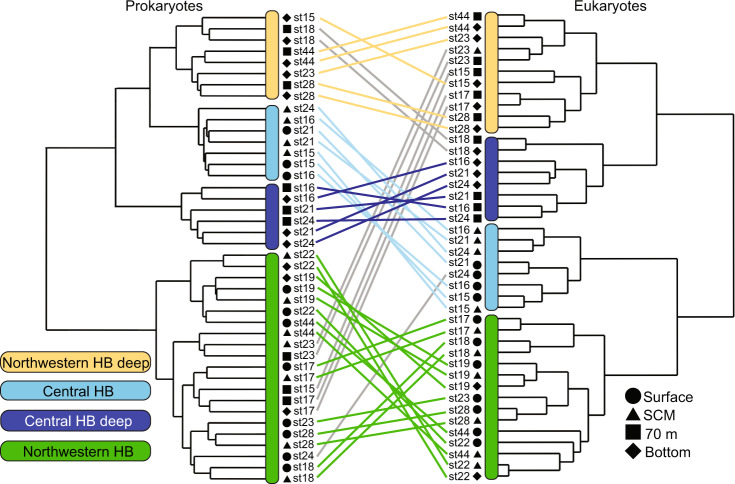
Microbial community structure. The dendrograms were constructed with Bray–Curtis distance of the 44 samples using the “ward.D2” method: prokaryotes (left tree) and eukaryotes (right tree). Symbols at the leaf extremities indicate depth category. Lines between the dendrograms show corresponding eukaryote and prokaryote placements from the same samples. The line color corresponds to designated environmental clusters when clustering is congruent. Grey lines indicate divergence of categories between eukaryotes and prokaryotes.

The hierarchical clustering analysis based on the Bray–Curtis distance showed a correlation between the structure of eukaryote (18S) and the prokaryote (16S) communities in rDNA (Fig. 2) and rRNA datasets (Supplementary Fig. S4). Biogeographic patterns in both upper and deeper waters were evident, with the northwestern and central HB clearly separated from each other. Both Procrustes and Mantel tests indicated that the microbial community structure was highly similar between rDNA and rRNA results (Supplementary Table S2). We then used the rDNA data to track the identity of potential sinking particles down the water column. The hierarchical dendrograms distinguished 4 clusters (Fig. 2). Deeper samples (bottom and 70 m) from northwestern HB and the Narrows (see Fig. 1) formed a single cluster, whereas deep central HB samples clustered apart. Surface and SCM samples from central HB and the Narrows formed a third cluster and surface and SCM samples from northwestern HB formed a fourth cluster. This fourth cluster also included deeper samples of the shallow more inshore stations (st22 and st19). To test for consistency and robustness of the clustering between eukaryotes and prokaryotes, the same analysis was conducted using the GUnifrac distance (Supplementary Table S2; Fig. S5). The GUnifrac and Bray–Curtis clustering using rDNA tended to be highly similar for prokaryotes (m^2^ = 0.14; Mantel R^2^ = 0.85) but slightly different for eukaryotes (m^2^ = 0.49; Mantel R^2^ = 0.84), with surface and SCM communities clustering together for eukaryotes using GUnifrac.

### Environmental influences on microbial assemblages

**Fig. 3:**
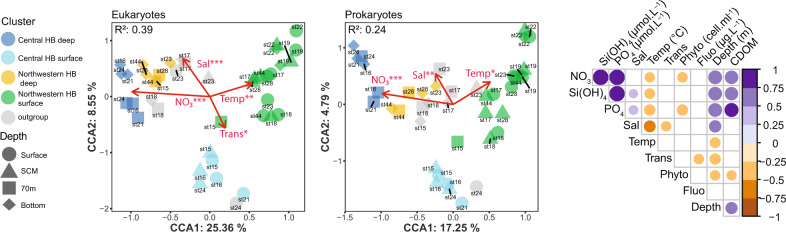
Ordination plots of distance-based redundancy analysis (db-RDA) and Pearson correlation between environmental variables of the stations. Microbial eukaryotes (left panel) and prokaryotes (central panel) communities. Only statistically significant environmental variables are shown. Symbol colors were defined according to the clusters identified in the hierarchical Bray–Curtis analysis from Fig. 2. Symbols indicate depth category. The right panel shows significant correlations between variables. Transmission abbreviated to “Trans” is a measure of the fraction of light that is absorbed or scattered.

To compare the explanatory power of environmental variables in structuring microbial communities, a distance-based redundancy analysis (db-RDA) was carried out using rDNA data (Fig. 3). The surface and SCM samples clearly separated from the deeper samples (70 m and bottom) along the first RDA axis, explaining 27.08% and 18.22% of the total variance respectively for eukaryotes and prokaryotes. The eukaryote and prokaryote analysis showed very similar trends corresponding to higher nutrient concentrations in the deep waters compared to the surface. At equal depths, higher nutrient concentrations in the center of the HB segregated samples along the first RDA axis. In the euphotic zone, northwestern HB samples were associated with warmer and saltier open waters compared to the ice-covered waters of central HB. Higher concentrations of pico‒phytoplankton in the ice-covered waters of central HB explained most of the variation along the secondary axis.

### Microbial community composition

**Fig. 4:**
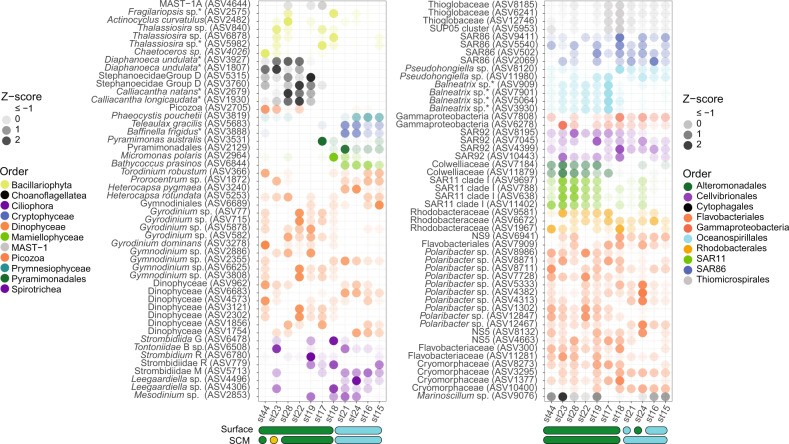
Heatmap of the 50 most abundant ASVs at surface and SCM layers. For each ASV, the Z-score shows the deviation from the mean relative abundance (Z-score = ASV relative abundance − mean relative abundance/standard deviation). The fill color of the circles corresponds to order level classifications. The color shapes at the bottom of the figure show clusters from Fig. 2.

In the rDNA dataset, z-scores calculated for the most abundant ‘top’ 50 eukaryotic and prokaryotic ASVs at the surface and SCM revealed a species-specific pattern differentiating samples from ice free waters in northwestern HB and ice-covered waters in central HB and the Narrows (Fig. 4). At the surface and SCM, these ASVs represented an average of 66.4 ± 9.8% of the total ASVs for eukaryotes and 40.2 ± 6.8% for prokaryotes. For eukaryotes, samples from central and northern HB (st17 and st18) showed higher relative abundance of 18S rDNA reads from small photosynthetic taxa. Particularly, *Phaeocystis pouchetti* (ASV 3819), *Micromonas polaris* (ASV 2964) and *Bathycoccus prasinos* (ASV 6844), which increased toward central HB and the Narrows, accounting for 6.5% of all ASVs. A higher relative abundance of diatoms was observed in northern HB at st17 and st18, with more reads associated with *Thalassiosira*. At the species level, *Fragilariopsis* sp. (ASV 2575) and *Actinocyclus curvulatus* (ASV 2482) reads were seen at relatively high abundance levels in northwestern HB but were almost absent from the ice-covered central HB. A clear spatial variability was detected between northwestern HB and central HB for the choanoflagellates *Diaphanoeca undulata* (ASV 1807 and 3927), *Calliacantha natans* (ASV 2679) and *Calliacantha longicaudata* (ASV 1930) and the dinoflagellate *Gyrodinium* (ASVs 715, 5878, 77 and 582), which had greater proportions in northwestern HB with 18.9% of the ASVs, compared to in Central HB with 2.6% of the ASVs. Maximum relative abundance of choanoflagellates was recorded at open water st22 and st28, where they represented more than 18% of the total reads.

Although less marked, a shift in composition from ice-covered to open water was also detected in the prokaryotic communities (Fig. 4). ASVs related to *Balneatrix* (ASVs 909, 7901, 5064 and 3930), SAR11 clade Ia (ASVs 9697, 788, 638 and 11402), unclassified *Colwelliaceae* (ASVs 7184 and 11879) and *Polaribacter* (ASVs 8986, 7728, 1302, 12847) were more abundant in the open water of northwestern HB. By contrast, ASVs affiliated to *Pseudohongiella* (ASVs 8120 and 11980), SAR86 (9411, 5540, 502, 2069) and Flavobacteriaceae NS9 (ASV 6941) were more abundant in Central HB, together representing 2.7% of the reads. Several representatives of SAR92 also showed different preferences for the open water (ASVs 8195, 10443) or ice-edge (ASVs 7045, 4399) conditions.

**Fig. 5:**
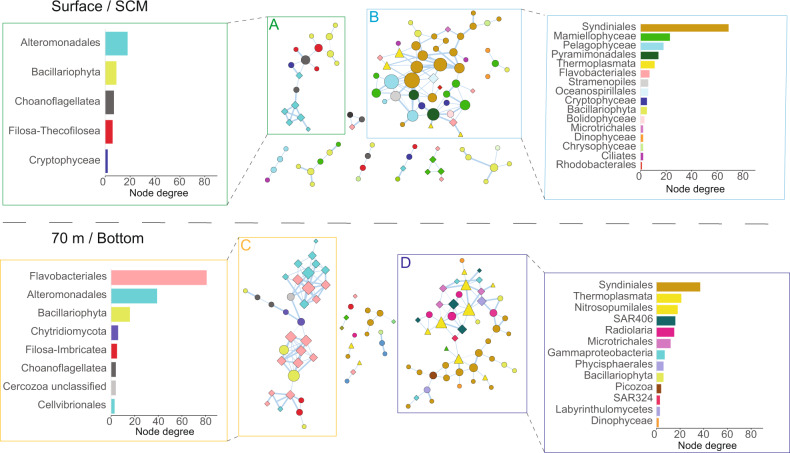
Co-occurrence network analysis calculated on rDNA. The size of the nodes is proportional to the connexion degree and the size of the edges are proportional to the number of methods that supported the association of two nodes. The shape of the nodes indicates the following domain: Eukaryotes (circles); Archaea (triangles); Bacteria (diamonds). The barplots show the node degree distribution of each taxonomic group in the corresponding frame, with surface and SCM (upper section) and 70 meters and bottom samples (lower section). The framed boxes correspond to the regional clusters; central HB (**A**); Northwestern HB (**B**); deep Northwestern HB (**C**); deep central HB (**D**). Networks outside the framed boxes are smaller subnetworks, which are for information only.

At 70 m and bottom depths in the rDNA dataset, the top 50 ASVs accounted for an average of 60.6 ± 11.1% of the total ASVs for eukaryotes and 62.4 ± 15.2% for prokaryotes (Supplementary Fig. S6). At these depths, pelagic diatoms such as *Thalassiosira* (ASVs 840, 6878, 5450 and 5631) and *Chaetoceros* (ASV 4026) were relatively more abundant in northwestern HB, together reaching up to 15.7% of the ASVs. Conversely, reads associated to Radiolaria and Syndiniales showed an increase in deeper central and northern Hudson Bay stations (Supplementary Fig. S6, S7). For Bacteria, deep waters of northwestern HB appeared favorable to *Polaribacter* (7.2%), *Nitrincolaceae* (3.5%) and *Colwellia* (0.9%). The potential ammonia oxidizers *Candidatus* Nitrosopumilus and Thermoplasmata group II and III were among the highest connected nodes in deep central HB and accounted for more than 15% of the total community in the rDNA and rRNA dataset (Fig. 5, Supplementary Figs. S6, S8).

### Co-occurrence network structure and composition

**Fig. 6:**
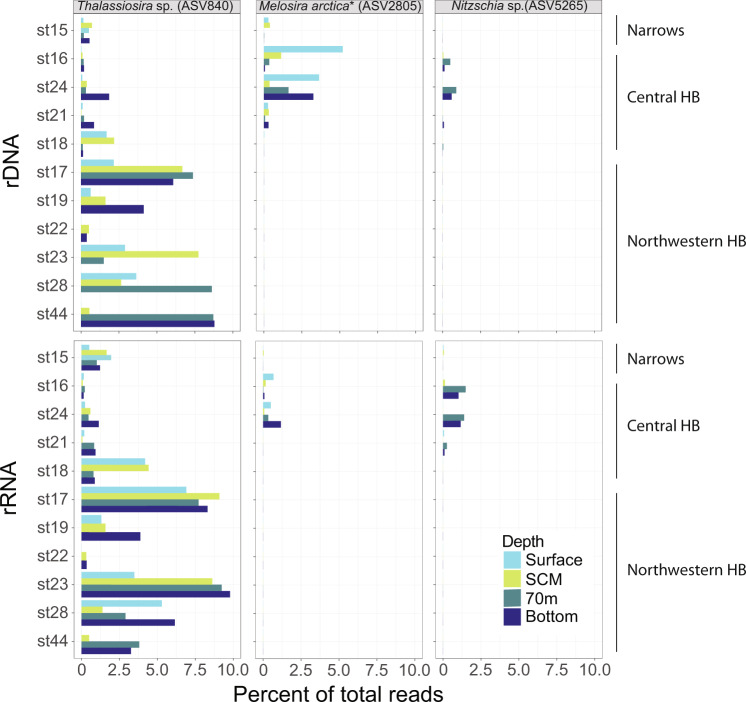
Distribution of key diatom ASVs. *Thalassiosira* sp., *Melosira arctica* and *Nitzschia sp*. down the water column from the rDNA (upper panels) and rRNA (lower panels).

Significant and robust associations were detected in the two subnetworks (Fig. 5, Supplementary Table S3). Edges retained for the final networks had high correlation scores for all four methods used, with Pearson >0.9, Spearman >0.87, mutual information >0.61 and Bray–Curtis distance <0.2. The average number of neighbors and the network density, which are two proxies for network connectivity were higher for the 70m-bottom network. Conversely, the network heterogeneity, which reflects the presence of hub nodes in the network was higher for the surface-SCM networks. The distribution of the relative abundance of nodes in the four major subnetworks reflected the regional hierarchical clustering (Supplementary Fig. S9). Nodes affiliated to *Colwellia* sp., Bacillariophyta and choanoflagellates were the most connected nodes in the northwestern HB subnetwork. In the central HB subnetwork, Syndiniales and Mamiellophyceae (*Micromonas* spp. and *Bathycoccus* spp.) represented most of the connected nodes. In the deep northwestern HB, *Polaribacter* sp., *Colwellia* sp. and Bacillariophyta had the highest node degree and Syndiniales, Thermoplasmata group II and III and Nitrosopumilales were the most connected nodes in deep central HB. The vertical repartition of the diatom nodes *Thalassiosira* (ASV 5450) and *Melosira arctica* (ASV 2805) assessed with rDNA but also found in rRNA showed that these taxa were present at all sampled depths (Fig. 6).

## Discussion

### Indirect influence of sea ice coverage on phytoplankton assemblages

During the BaySys study in late spring 2018, based on in situ phytoplankton parameters, there was a probable under-ice bloom dominated by diatoms in central HB [13, 14]. In contrast, at the ice-edge, nutrient data and our results suggest that the low nitrate concentrations favored a pico-phytoplankton dominated community (Figs. 3, 4, Supplementary Fig. S1, S2). Small photosynthetic genera such as *Bathycoccus* and *Micromonas* often dominate under low nutrients conditions in the Arctic, as their smaller surface-to-volume ratio allow them to outcompete diatoms [9, 47]. The pico-phytoplankton community at the ice edge is consistent with sampling after an under-ice bloom, when nutrients would have already been consumed.

In the ice-free northwestern HB (st18, st23, st28 and st44), the earlier spring bloom would also have depleted surface nutrients and led to formation of an SCM below the pycnocline where nutrient concentrations remained high but still well within the euphotic zone. The higher nutrients favored larger phytoplankton, including diatoms (Figs. 1, 4). While reads from *Micromonas, Bathycoccus* and *Phaeocystis* were correlated with the pico-phytoplankton size fraction and, to a lesser extent, photosynthetic nano‒flagellate reads were correlated in surface waters to the FCM counts in the phytoplankton category, there was not a good relationship with diatoms. The lack of correlation with FCM counts and diatoms would be due to the limitations of our flow cytometry system (Supplementary Fig. S3, Table S1). The fast rate used for the phytoplankton results in 22 µm core size, which is optimum for nano‒plankton (3–20 µm) but not for larger diatoms such as *Actinocyclus* and *Thalassiosira* spp., which would explain the differences between flow cytometry and molecular analysis (Table S1).

Ice cover influences microbial assemblages in the water column predominantly by decreasing light availability, and during sea ice melt, by freshening the surface and contributing to increased stratification. As light becomes available, nutrients in the stratified surface are quickly drawn down [48, 49]. Other factors, such as the freshwater inputs from river runoff and distance from shore, also influence stratification and can add some nutrients to surface waters, contributing to higher phytoplankton production at the time of sampling [14]. Our survey of the dominant microbial communities, although only a snapshot and not an in-depth analysis of seasonal succession, captured differences in phytoplankton assemblages between central and northwestern HB that were consistent with the spatiotemporal pattern of sea-ice.

### Cascading effect of phytoplankton-derived OM on heterotrophs

Cascading changes in heterotrophic communities have been linked to the abundance and composition of phytoplankton during or after blooms [22, 50]. To test for similar cascading effects, we carried out co-occurrence network analysis, which revealed that biotic interactions were a determinant factor for community structure in HB. The structure of association networks can provide deep insights into the organization of microbial communities and be used to identify ecological units where indicator taxa co-occur in response to shared niches [51–53]. Most of the links in the central HB network involved group I and II Syndiniales ASVs and phytoplankton such as *Micromonas*, pelagophytes and *Fragilariopsis* (Fig. 5b). Syndiniales have a parasite lifestyle and can infect a broad range of organisms from other protists to fishes [54]. The high connectivity of Syndiniales in co-occurrence networks was previously reported in the global ocean and highlighted the role of dinoflagellate parasites as top down effectors of phytoplankton population structure [55, 56]. Recent studies in the Southern Ocean indicated that Syndiniales group I can become super-abundant at the ice-edge [57]. In keeping with Syndiniales being metabolically active, we found Syndiniales ASVs in both rDNA and rRNA in central HB [58, 59] at the ice-edge (Supplementary Fig. S7). The high connectivity of Syndiniales nodes with picophytoplankton and *Fragilariopsis* ASVs would be consistent with a role in the collapse of under-ice blooms, where they could act in synergy on multiple species. The numerous edges connecting Syndiniales ASVs with other Syndiniales in our network analysis at the ice edge suggests co-infections of the same host by diverse Syndiniales, and could explain the correlations [55]. Kellog et al. [60] also reported that Syndiniales OTUs co-occurred with a broad range of protists including other Syndiniales in the coastal Beaufort Sea.

The open water in northwestern HB appeared favorable for choanoflagellates (Figs. 4, 5a), which prey on bacteria and are consumed by zooplankton; moving carbon and nutrients to higher trophic levels [61, 62]. Choanoflagellates are diverse [63, 64] and can reach high concentrations in polar seas in response to high biomass from primary and secondary production [65, 66]. Here, choanoflagellate ASVs were correlated with Gammaproteobacteria ASVs, suggesting a direct response to bacterial food sources.

The various bacterial communities were associated with temperature, salinity and nitrate, but low r^2^ coefficients in the db-RDA suggests much less influence, compared to the associated protists including phytoplankton (Fig. 4). The quality and quantity of organic carbon produced and released by phytoplankton varies providing a series of ecological niches for bacterial communities [28, 67]. At the ice-edge, the bacterial community was dominated by *Pseudohongiella*, Flavobacteriaceae, SAR92 representatives and SAR86 lineages. These lineages are frequently found during phytoplankton blooms, recycling phytoplankton-derived OM. These lineages have bacteriorhodopsins, and are efficient in the light compared to non-bacteriorhodopsin lineages [68–71], which is consistent with being nearer the surface. By contrast, the relative abundance of ASVs of some members of the *Colwelliaceae*, which is also associated with decaying spring blooms or ice-algal aggregates [72, 73], increased in the open waters and were major nodes in the northwestern HB network (Fig. 5). Although they were not represented in the subnetwork, *Balneatrix*, a genus in the Gammaproteobacteria, was found in the northwestern HB samples (Fig. 4). *Balneatrix* is a frequent particle-associated bacteria succeeding blooms in polar coastal communities [74, 75], suggesting that these bacterial lineages were more adapted to open water conditions or OM derived from the larger phytoplankton in the SCM. Interestingly, the shift in bacterial community composition between the ice-edge and open water stations was not accompanied by any change in bacteria cell abundance (supplementary Fig. S1) in keeping with the high number of bacterial grazers.

### Sinking particles and decaying OM from sea ice bloom in deep communities

A fundamental question in microbial oceanography is how surface processes affect the composition of the microbial communities at depth. The HB is relatively shallow (~125 m) compared to other seas, and taxa from the surface could sink to depth and influence deeper microbial assemblages. The network complexity was high in the deeper waters, having the greatest numbers of nodes and edges as well as highest density and node degree, and lower heterogeneity (Supplementary Table S3). The increase in network size and complexity with depth could be interpreted as increased community organization and interactions. There is limited water exchange between deep HB and surface waters and a long residence time of deep water between 4 to 14 years [76] would provide stable environmental conditions sufficient for stochastic processes to dominate and shape complex and interactive food webs as reported elsewhere [19, 77]. Deep-water samples separated into two ecological niches with communities from the ice-covered central HB (st16, st21 and st24) differentiated from ice-free stations (st44, st23, st28, st18) and st15 in the Narrows (Fig. 5, Supplementary Fig. S6), consistent with species enrichments from the immediate overlying water column.

The pelagic diatoms *Chaetoceros* and *Thalassiosira* were detected in the deep and low light waters of Northwestern HB (Supplementary Fig. S6). Moreover, *Thalassiosira* sp. (ASV 840), was detected at all depths through the water column in the rDNA and rRNA datasets of northwestern HB (Fig. 6). The co-occurrence network indicated that this diatom node was strongly associated with several heterotrophic bacteria related to *Colwellia* and *Polaribacter* that have been reported on sinking particles of senescent phytoplankton in deep marine systems [72, 78] (Fig. 5c). These results suggest an export of the surface primary production to the deeper water through sinking particles of pelagic diatoms that bloomed after the ice melt. The proportion of large diatoms and chain forming diatoms may have been underestimated because we prefiltered through a 50 µm mesh. The taxonomic composition of the largest aggregates and microbial eukaryotes living on marine snow is similarly uncertain.

In deep central HB, the most connected nodes were predominantly associated with Syndiniales group II and members of the Radiolaria, especially from the Order Chaunacanthida (Fig. 5d), both of which, can be major contributors to export flux to the deep ocean [79]. Direct endoparasitic interactions between Syndiniales group II and Radiolaria from the order Spumellaria and Nassellaria have been detected using single cell sequencing [80, 81]. As species from the order Chaunacanthida can produce reproductive cysts that sink rapidly from the surface to deep waters [82], it is likely that the co-occurrence of Chaunacanthida with Syndiniales in the deep network represents a potential parasitic interaction. Several bacteria, such as Sva0996, or the heterotrophic sulfur oxidizing SAR406 (Marinimicrobia) and SAR324 taxa were detected in the deep central HB subnetwork. Although these lineages are frequently reported from sub-oxic mesopelagic environments [83–85], the central HB water column was well oxygenated from the surface to the bottom (Supplementary Table S1). The occurrence of these taxa might be explained by oxygen-limited microhabitats within sinking particles [86]. These particle-associated microbes have also been recorded in sediment traps as deep as 4000 m, in association with Syndiniales and Rhizaria [78]. The results are consistent with the sinking particles creating a persistent microhabitat in central HB that favors the association between the two protist groups.

Interestingly, the sympagic diatoms *Nitzchia* sp. and *M. arctica* were evident in the deep central network, and *M. arctica* was detected throughout the water column, but exclusively in the ice-covered central HB (Fig. 6). The release of *NItzchia* cells from sea ice has been reported in HB during ice breakup where they contributed to the water column assemblages [87]. *Melosira* was estimated to be an important contributor to the under-ice production during late spring in 2017 in central HB [14]. *Melosira* attaches to the ice bottom and forms visible strands on under ice surfaces. In the central Arctic Ocean, *Melosira* is estimated to contributes >45% of total primary production and >85% of carbon export to the deep central Arctic (>4000 m), arriving nearly intact on the sediment surface [88]. The detection of these sympagic cells at depth in HB is consistent with a release of these algae prior sea ice breakup, and on sinking to the bottom, would provide carbon substrate directly to benthic fauna and bacteria.

The high connectivity and relative abundance of potential archaeal ammonia oxidizers highlighted the structuring role of these organisms in deep central HB (Fig. 5, Supplementary Fig. S6, S8). Considering that the universal primers used in this study are not specifically designed to target Archaea, the relative abundance of Archaea here was probably underestimated [89]. The accumulation of inorganic nutrients at depth in the center of the bay is attributed to a combination of physical transfer from rivers and decomposition of OM supplied by diatom export [90]. An injection of riverine nitrate into the deep layer cannot be considered as the dominant process as nitrate at the surface is usually rapidly consumed by primary producers. Although nitrification processes cannot be directly inferred from our metabacoding dataset, our results support the hypothesis that at least some of the nitrate pool detected in deep HB might be due to nitrification by Archaea.

## Conclusion

In this study, we demonstrated through a co-occurrence network approach that sea ice indirectly affects microbial community structure from the surface to the deep HB. As model predictions in HB suggests earlier ice breakup and longer open water periods, our results support the scenario of Wassmann & Reigstad [91], that lengthening of the open water season should increase the period dominated by regenerated production achieved by heterotrophic protists and bacterial degraders. Longer open water would also increase the contribution of diatoms from the SCM to the deep OM export, which would impact deep communities that rely on algal deposition. The efficiency of this diatom dominated SCM to fix and retain atmospheric CO_2_ would depend on its vertical position in the water column [49]. It is likely that a lengthening of this open water scenario would affect the fate of OM in the HB and favor the tendency for HB to be a source of rather than a sink for atmospheric CO_2_. Altogether, these results highlight the importance of monitoring all the components of microbial food web to better understand changing ecosystem function in the Arctic Ocean.

## Supplementary information

Supplementary file

Table S1

Table S2

Table S3

## Data Availability

Sequence Data has been deposited in NCBI under BioProject accession numbers PRJNA627250 and PRJNA721720 for eukaryotes and prokaryotes, respectively. Metadata and Reports on the Hudson Bay System Study (BaySys) are available at https://dev.uni-manitoba.links.com.au/data/project/baysys.
