# Supplementary material for: Contrasting sea ice conditions shape microbial food webs in Hudson Bay (Canadian Arctic)
Source: ISME Commun. 2022 Oct 23;2:104. doi: 10.1038/s43705-022-00192-7 (PMC9723562; doi:10.1038/s43705-022-00192-7)
Supplement: Supplementary file 1 — Supplementary file [file 43705_2022_192_MOESM1_ESM.pdf]

Supplementary online Material: ISME Communications (submitted)

“Contrasting sea ice conditions shape microbial food webs in Hudson Bay (Canadian Arctic).”

By: *Loïc Jacquemot, Adrien Vigneron, Jean-Éric Tremblay and Connie Lovejoy*

Table S1. meta data. station (STN); Depth category (Feature, as in main text); Sea Ice Concentration (SIC); Salinity (Sal); Temperature (TEMP); Nitrate (NO3); Nitrite (NO2); Silicate (Si(OH); Phosphate (PO4); Transmission (Trans); Colored Dissolved Organic Matter (CDOM); Chlorophyll Fluorescence (CHL FLUO): Oxygen (O2).

Table S2. Procrustes and Mantel test statistic scores. First score represents m2 statistic which is based on the sum of the squared division of procrustes analysis. The second number is the Mantel test r statistic score based on 999 permutations. All scores were significant ($p<0.001$). Bray-Curtis (BC); GUnifrac (GU).

Table S3. Network and subnetwork statistics: from the Fig. 5, main text.

Figure S1. Enumeration of phytoplankton and bacterial cells using flow cytometry. Nano-phytoplankton, pico-phytoplankton and cyanobacteria are gated using chlorophyll *a* (FL3), phycoerythrin (FL2) and forward-scattered light (FSC) lasers (A). Bacteria are gated using chlorophyll *a* (FL3) and Sybr green (FL1) (B). Barplots represents cell concentrations at the surface and SCM (C).

Figure S2. Rank abundance curve of prokaryotes and microbial eukaryotes. Top 500 representatives of each group were retained for co-occurrence network analysis.

Figure S3. Flow cytometry measurements of phytoplankton. Pico-phytoplankton (left) and nano-phytoplankton (middle and right) size classes versus relative abundance of phytoplankton ASVs reads. Nanoflagellates grouped the Orders; Cryptophyceae, Pyramimonadales, and small Bacillariophyta, Bolydophyceae, Chrysophyceae and Pelagophyceae. Pvalue : *** < 0.001.

Figure S4. Microbial community structure dendrograms. The dendrograms were constructed using the rRNA dataset with Bray-Curtis distance of the 44 samples using the "ward.D2" method: prokaryotes (left tree) and eukaryotes (right tree). Symbols at the leaf extremities indicate depth category. Lines between the dendrograms show corresponding eukaryotes and prokaryotes from the same samples. The line color corresponds to designated environmental clusters when clustering is congruent. Grey lines indicate divergence of categories between eukaryotes and prokaryotes.

Figure S5. NMDS analysis on eukaryotes and prokaryotes. Comparison of Bray-Curtis and GUnifrac distances. The analysis has been conducted with rDNA samples.

Figure S6. Heatmap of the 50 most abundant ASVs at 70 meters and bottom. For each ASV, the Z-score shows the deviation from the mean relative abundance (Z-score = ASV relative abundance – mean relative abundance / standard deviation). The color fill of the circles corresponds to order level classifications. The color shapes at the bottom of the figure show clusters from the Figure 2.

Figure S7. Percent of reads affiliated to Syndiniales in rDNA and rRNA datasets.

Figure S8. Percent of reads affiliated to Archaea in rDNA and rRNA datasets.

Figure S9. Barplot of cumulative relative abundance of the nodes as identified in the co-occurrence network analysis (Fig. 5). Each panel represents corresponding regional clusters; Northwestern HB (A), central HB (B), Northwestern HB deep (C), central HB deep (D).

Table S1. meta data. station (STN); Depth category (Feature, as in main text); Sea Ice Concentration (SIC); Salinity (Sal); Temperature (TEMP); Nitrate (NO3); Nitrite (NO2); Silicate (Si(OH); Phosphate (PO4); Transmission (Trans); Colored Dissolved Organic Matter (CDOM); Chlorophyll Fluorescence (CHL FLUO): Oxygen (O2).

STN	DATE	FEATURE	DEPTH (m)	SIC (%)	SAL	TEMP (°C)	NO3 (µmol L^{-1})	NO3 (µmol L^{-1})	SIOH (µmol L^{-1})	PO4 (µmol L^{-1})	TRANS	CDOM	CHL FLUO (µg L^{-1})	O2 (µmol kg^{-1})
st15	05/06/2018	Surface	0	50	31.8	-1.5	4.19	0.04	9.44	0.89	97.93	1.76	0.09	312
st16	06/06/2018	Surface	0	97	31.5	-1.5	3.10	0.08	8.12	0.83	98.84	1.77	0.11	322
st17	07/06/2018	Surface	0	3	32.4	-0.7	0.52	0.03	5.25	0.66	93.49	1.12	0.42	345
st18	08/06/2018	Surface	0	27	32.2	-0.7	0.04	0.02	5.48	0.56	94.42	1.36	0.69	349
st19	09/06/2018	Surface	0	7	32.9	-0.4	0.00	0.03	0.00	0.56	96.14	1.51	0.12	347
st21	10/06/2018	Surface	0	97	31.5	-1.3	1.03	0.06	4.56	0.67	97.15	na	0.16	333
st22	11/06/2018	Surface	0	3	31.1	2.4	0.00	0.01	0.00	0.53	93.67	2.25	0.09	334
st23	11/06/2018	Surface	3	3	32.6	0.2	0.00	0.04	0.00	0.45	93.27	1.44	1.41	360
st24	12/06/2018	Surface	0	20	31.1	-1.3	0.43	0.05	3.16	0.65	96.40	1.66	0.26	338
st28	15/06/2018	Surface	0	3	32.3	1.2	0.00	0.00	0.17	0.42	95.18	1.16	0.16	355
st44	24/06/2018	Surface	0	3	30.1	2.0	1.61	0.00	1.31	0.50	98.32	1.27	0.03	342
st15	05/06/2018	SCM	33	50	32.0	-1.7	4.07	0.05	9.66	0.88	97.76	1.81	0.27	313
st16	06/06/2018	SCM	20	97	31.9	-1.7	3.75	0.08	8.77	0.86	98.66	1.91	0.30	317
st17	07/06/2018	SCM	15	3	32.6	-1.5	1.58	0.05	6.81	0.78	92.91	1.87	0.95	322
st18	08/06/2018	SCM	20	27	32.4	-1.1	0.22	0.01	5.65	0.35	93.05	1.50	3.05	345
st19	09/06/2018	SCM	35	7	32.9	-1.0	0.00	0.03	0.00	0.58	95.96	1.53	0.77	344
st21	10/06/2018	SCM	30	97	31.9	-1.6	3.23	0.09	6.51	0.82	97.74	1.59	0.71	327
st22	11/06/2018	SCM	38	3	33.2	-1.0	0.00	0.01	0.00	0.64	96.34	1.55	0.53	345
st23	11/06/2018	SCM	50	3	32.8	-1.3	2.57	0.08	5.14	0.84	92.16	1.66	2.73	318
st24	12/06/2018	SCM	42	20	31.5	-1.4	2.18	0.09	5.56	0.77	97.49	1.86	0.80	331
st28	15/06/2018	SCM	35	3	32.5	-1.3	1.34	0.01	3.30	0.68	89.61	1.65	4.81	320

st44	24/06/2018	SCM	37	3	32.6	-1.6	1.13	0.06	5.91	0.82	96.91	1.97	1.96	325
st15	05/06/2018	70m	69	50	32.4	-1.6	2.40	0.01	7.31	0.77	95.31	1.76	0.98	324
st16	06/06/2018	70m	69	97	32.4	-1.2	6.86	0.01	13.06	1.04	99.16	1.92	0.06	282
st17	07/06/2018	70m	70	3	32.8	-1.6	5.08	0.08	11.67	1.03	90.79	1.82	0.31	295
st18	08/06/2018	70m	70	27	33.3	-1.8	4.47	0.10	10.74	0.73	96.27	1.65	0.09	310
st21	10/06/2018	70m	70	97	32.6	-1.4	7.37	0.04	14.90	1.09	99.31	1.80	0.13	274
st23	11/06/2018	70m	70	3	32.9	-1.5	5.73	0.06	11.60	1.07	93.74	1.82	1.17	289
st24	12/06/2018	70m	70	20	32.6	-1.1	9.06	0.04	17.30	1.21	99.64	2.03	0.06	249
st28	15/06/2018	70m	70	3	32.7	-1.5	6.85	0.00	13.19	1.07	96.90	1.79	0.56	288
st44	24/06/2018	70m	70	3	32.8	-1.7	6.99	0.01	17.28	1.18	95.54	2.08	1.03	286
st15	05/06/2018	Bottom	175	50	32.6	-1.5	4.47	0.05	10.23	0.90	92.15	1.72	2.18	337
st16	06/06/2018	Bottom	124	97	32.7	-1.1	8.39	0.02	18.03	1.15	92.99	2.01	0.04	266
st17	07/06/2018	Bottom	80	3	32.9	-1.6	5.17	0.08	11.77	1.03	90.52	1.90	na	296
st18	08/06/2018	Bottom	102	27	33.4	-1.8	4.40	0.09	11.04	0.75	96.41	1.68	0.09	312
st19	09/06/2018	Bottom	70	7	33.0	-1.0	0.00	0.03	0.09	0.62	97.24	1.69	0.22	341
st21	10/06/2018	Bottom	137	97	33.0	-1.3	10.68	0.05	27.32	1.40	88.63	2.11	0.07	232
st22	11/06/2018	Bottom	55	3	33.2	-1.0	0.00	0.01	0.00	0.64	96.38	1.57	0.63	345
st23	11/06/2018	Bottom	100	3	33.1	-1.8	4.83	0.07	9.21	1.02	85.99	1.91	1.33	292
st24	12/06/2018	Bottom	175	20	33.0	-1.3	12.88	0.04	35.72	1.62	90.21	2.37	0.08	206
st28	15/06/2018	Bottom	150	3	33.2	-1.8	5.14	0.01	12.96	1.05	89.18	1.83	0.40	303
st44	24/06/2018	Bottom	90	3	32.9	-1.6	7.17	0.01	18.80	1.20	90.47	2.17	0.37	266

Table S2. Procrustes and Mantel test statistic scores. First score represents m2 statistic which is based on the sum of the squared division of procrustes analysis. The second number is the Mantel test r statistic score based on 999 permutations. All scores were significant ($p<0.001$). Bray-Curtis (BC); GUnifrac (GU).

		Euk				Prok			
		BC_DNA	GU_DNA	BC_RNA	GU_RNA	BC_DNA	GU_DNA	BC_RNA	GU_RNA
	BC_DNA	0 / 1	0.49 / 0.84	0.03 / 0.95	0.11 / 0.83	0.29 / 0.80	0.48 / 0.74	0.37 / 0.78	0.48 / 0.75
	GU_DNA		0 / 1	0.47 / 0.83	0.54 / 0.88	0.66 / 0.67	0.43 / 0.70	0.55 / 0.66	0.33 / 0.71
	BC_RNA			0 / 1	0.06 / 0.88	0.27 / 0.77	0.44 / 0.74	0.34 / 0.76	0.72 / 0.73
Euk	GU_RNA				0 / 1	0.21 / 0.74	0.35 / 0.79	0.25 / 0.71	0.39 / 0.76
	BC_DNA					0 / 1	0.14 / 0.85	0.04 / 0.94	0.23 / 0.84
	GU_DNA						0 / 1	0.07 / 0.86	0.05 / 0.93
	BC_RNA							0 / 1	0.12 / 0.89
Prok	GU_RNA								0 / 1

Table S3. Network and subnetwork statistics: from the Fig. 5, main text.

Cluster	Nodes	*Deg*	*Dens*	*H*	Edges	Ed_4	Ed_3	Ed_2	*r*	*ρ*	*MI*	*BC*
Surf/SCM	98	2.776	0.029	0.776	136	59	72	5	0.90	0.87	0.68	0.20
NW HB	17	2.47	0.15	0.52	21	10	8	3				
Central HB	49	3.59	0.08	0.73	88	38	48	2				
70m/bottom	100	3.34	0.03	0.69	167	72	77	18	0.93	0.89	0.61	0.18
NW HB	33	4.73	0.15	0.52	78	50	26	2				
Central HB	49	3.06	0.06	0.66	75	19	47	9				

deg is the mean degree or the average number of neighbors, *dens* is the network density, *H* is the network heterogeneity, Ed_4 is the number of edges validated by 4 methods, Ed_3 is the number of edges validated by 3 methods, Ed_2 is the number of edges validated by 2 methods, *r* is the mean Pearson coefficient, *ρ* is the mean Spearman coefficient, *MI* is the mean mutual information, *BC* is the mean Bray Curtis distance.

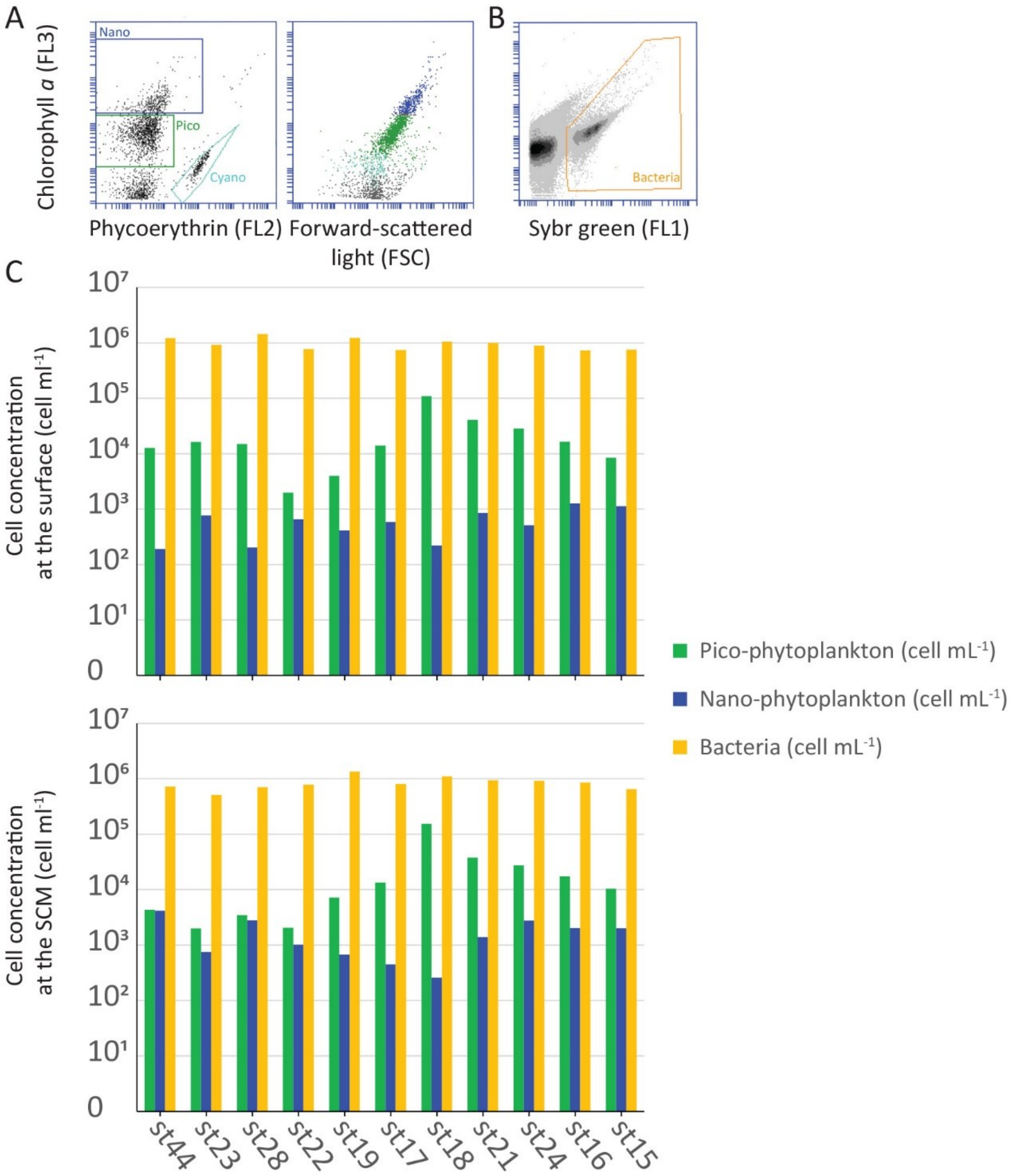

Figure S1. Enumeration of phytoplankton and bacterial cells using flow cytometry. Nano-phytoplankton, pico-phytoplankton and cyanobacteria are gated using chlorophyll *a* (FL3), phycoerythrin (FL2) and forward-scattered light (FSC) lasers (A). Bacteria are gated using chlorophyll *a* (FL3) and Sybr green (FL1) (B). Barplots represents cell concentrations at the surface and SCM (C).

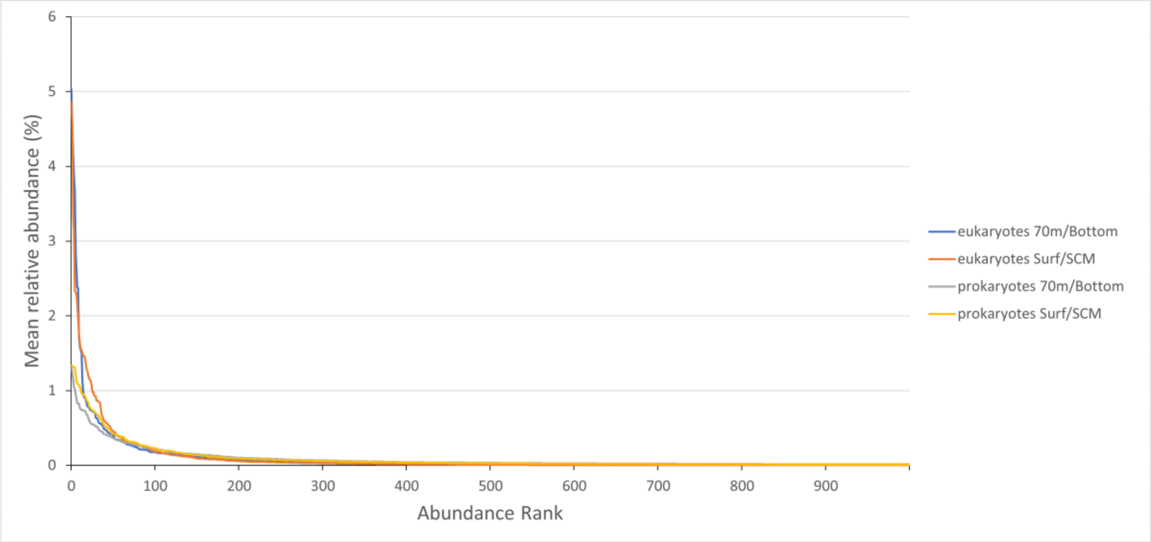

Figure S2. Rank abundance curve of prokaryotes and microbial eukaryotes. Top 500 representatives of each group were retained for co-occurrence network analysis.

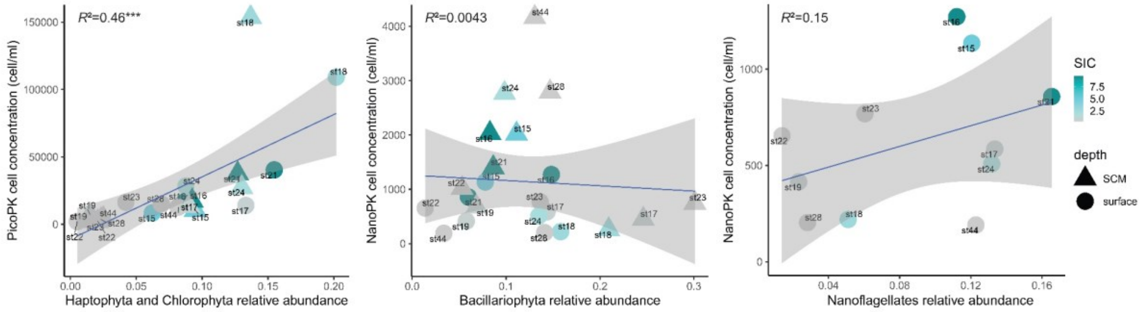

Figure S3. Flow cytometry measurements of phytoplankton. Pico-phytoplankton (left) and nano-phytoplankton (middle and right) size classes versus relative abundance of phytoplankton ASVs reads. Nanoflagellates grouped the Orders; Cryptophyceae, Pyramimonadales, and small Bacillariophyta, Bolydophyceae, Chrysophyceae and Pelagophyceae. Pvalue : *** < 0.001.

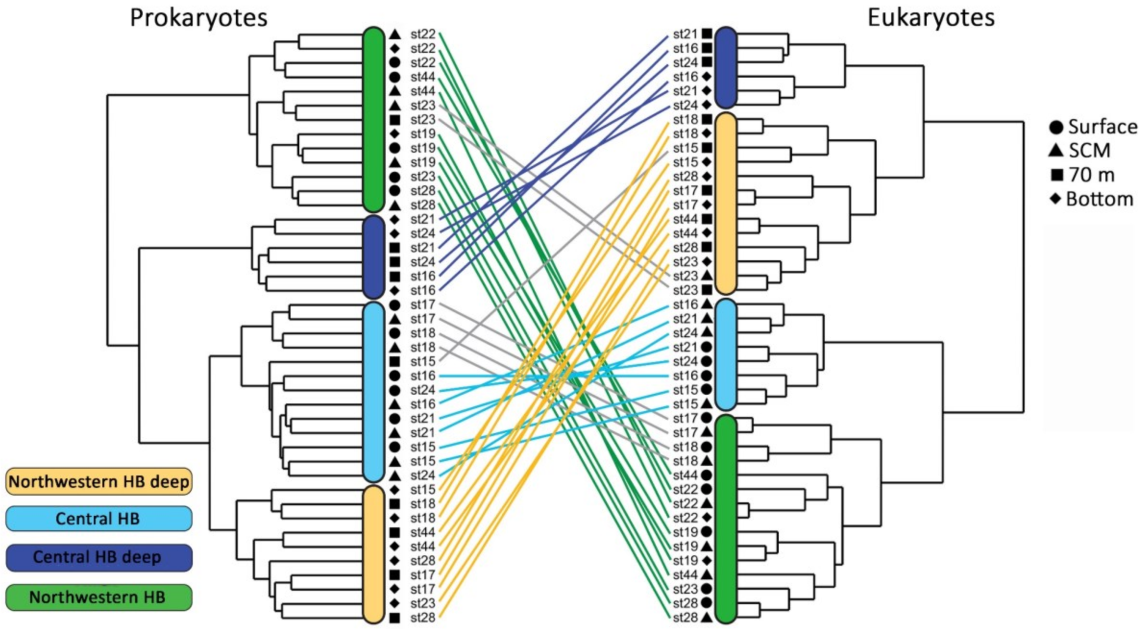

Figure S4. Microbial community structure dendrograms. The dendrograms were constructed using the rRNA dataset with Bray-Curtis distance of the 44 samples using the "ward.D2" method: prokaryotes (left tree) and eukaryotes (right tree). Symbols at the leaf extremities indicate depth category. Lines between the dendrograms show corresponding eukaryotes and prokaryotes from the same samples. The line color corresponds to designated environmental clusters when clustering is congruent. Grey lines indicate divergence of categories between eukaryotes and prokaryotes.

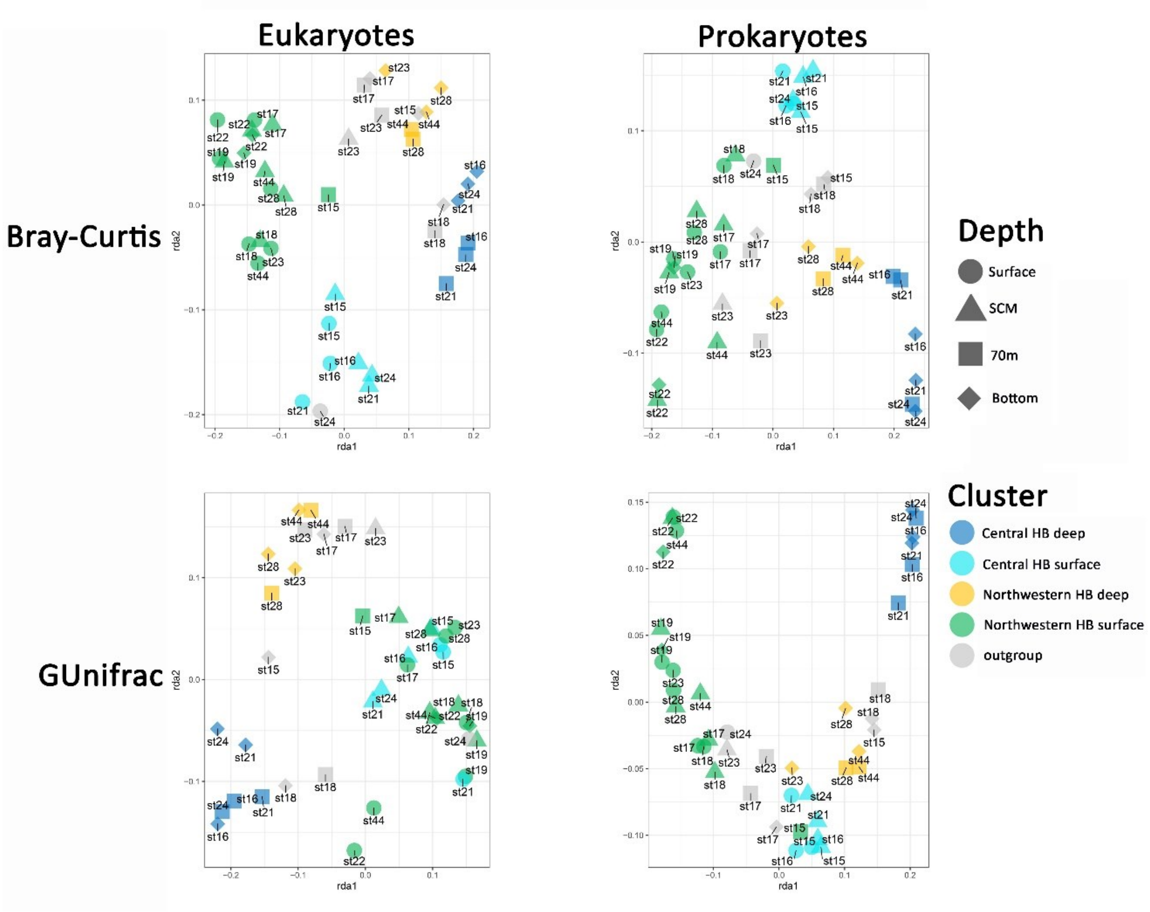

Figure S5. NMDS analysis on eukaryotes and prokaryotes. Comparison of Bray-Curtis and GUnifrac distances. The analysis has been conducted with rDNA samples.

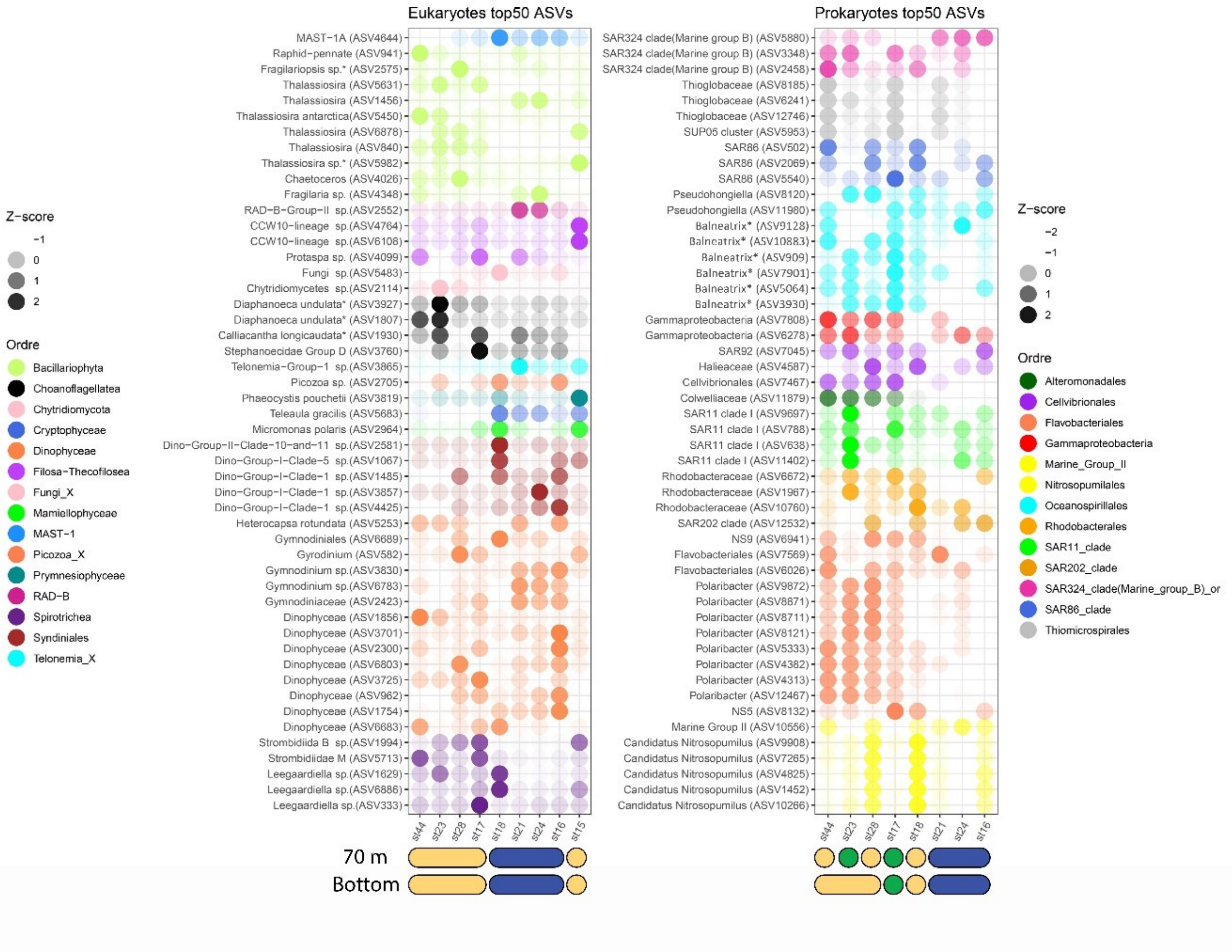

Figure S6. Heatmap of the 50 most abundant ASVs at 70 meters and bottom. For each ASV, the Z-score shows the deviation from the mean relative abundance (Z-score = ASV relative abundance – mean relative abundance / standard deviation). The color fill of the circles corresponds to order level classifications. The color shapes at the bottom of the figure show clusters from the Figure 2.

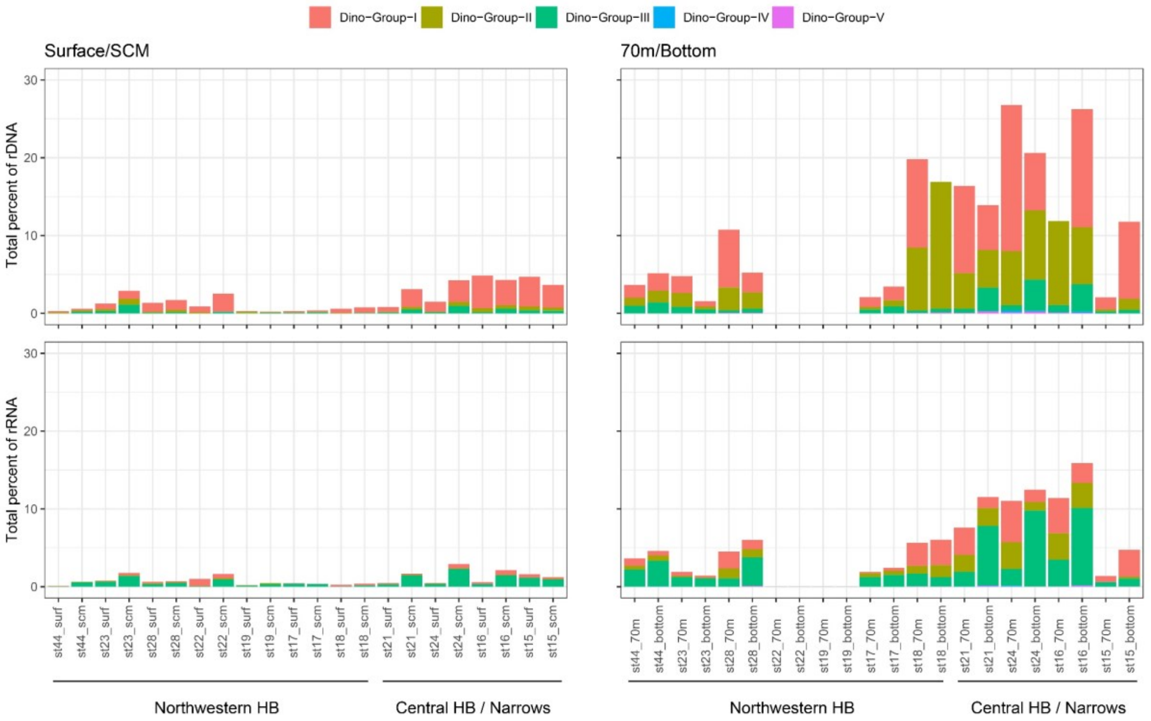

Figure S7. Percent of reads affiliated to Syndiniales in rDNA and rRNA datasets.

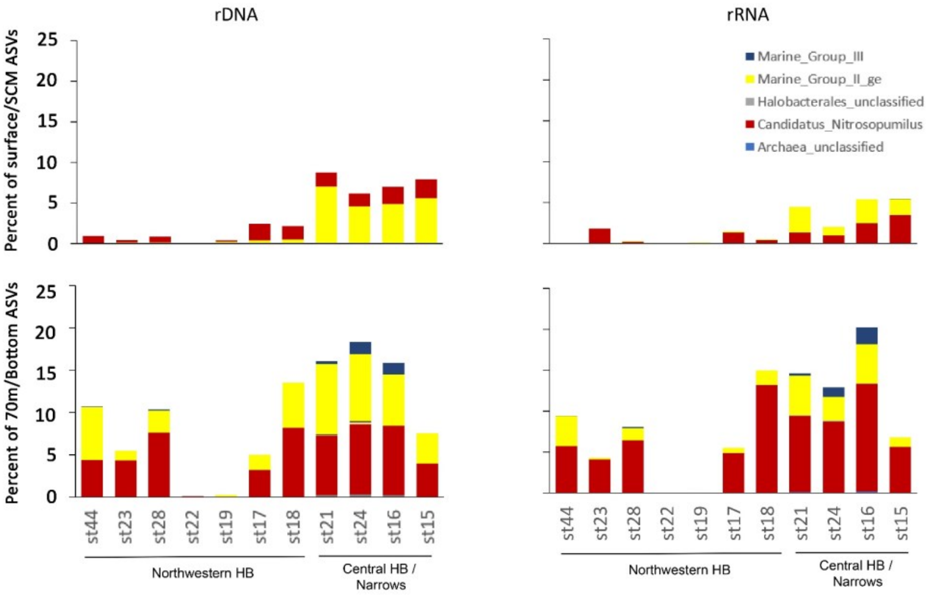

Figure S8. Percent of reads affiliated to Archaea in rDNA and rRNA datasets.

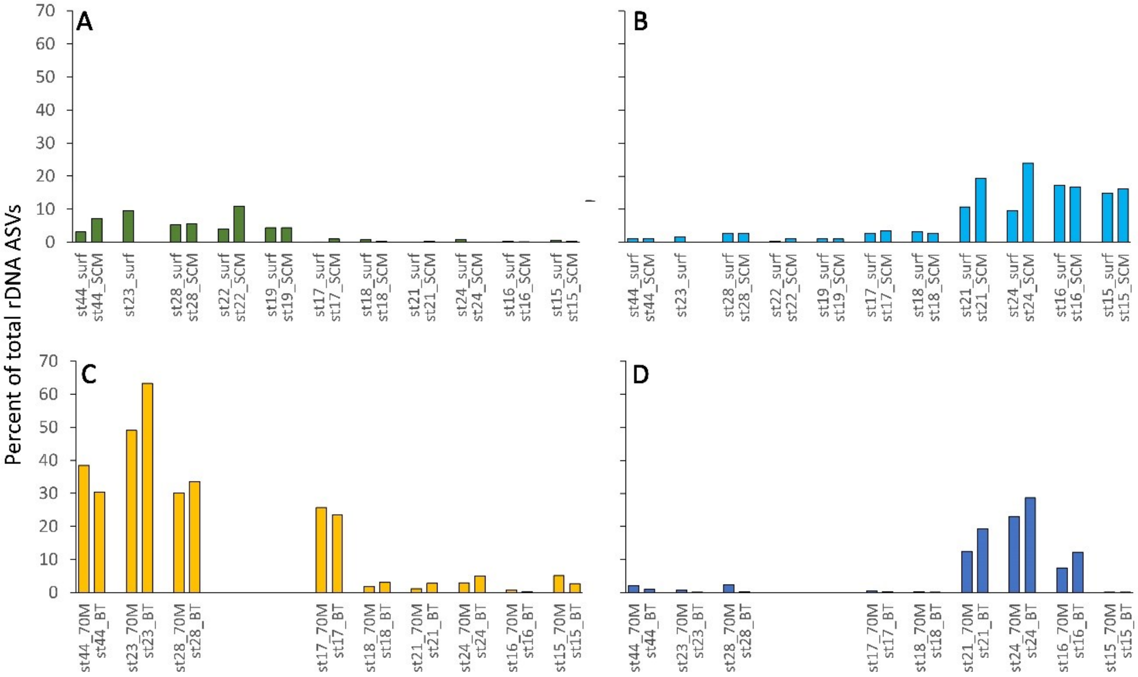

Figure S9. Barplot of cumulative relative abundance of the nodes as identified in the co-occurrence network analysis (Fig. 5). Each panel represents corresponding regional clusters; Northwestern HB (A), central HB (B), Northwestern HB deep (C), central HB deep (D).
